# Absence of XMRV and Closely Related Viruses in Primary Prostate Cancer Tissues Used to Derive the XMRV-Infected Cell Line 22Rv1

**DOI:** 10.1371/journal.pone.0036072

**Published:** 2012-05-16

**Authors:** Jaydip Das Gupta, Ka-Cheung Luk, Ning Tang, Christina Gaughan, Eric A. Klein, Eugene S. Kandel, John Hackett, Robert H. Silverman

**Affiliations:** 1 Department of Cancer Biology, Lerner Research Institute, Cleveland Clinic, Cleveland, Ohio, United States of America; 2 Abbott Diagnostics, Emerging Pathogens and Virus Discovery, Abbott Park, Illinois, United States of America; 3 Abbott Molecular, Des Plaines, Illinois, United States of America; 4 Glickman Urologic and Kidney Institute, Cleveland Clinic, Cleveland, Ohio, United States of America; 5 Roswell Park Cancer Institute, Buffalo, New York, United States of America; Burnet Institute, Australia

## Abstract

The 22Rv1 cell line is widely used for prostate cancer research and other studies throughout the world. These cells were established from a human prostate tumor, CWR22, that was serially passaged in nude mice and selected for androgen independence. The 22Rv1 cells are known to produce high titers of xenotropic murine leukemia virus-related virus (XMRV). Recent studies suggested that XMRV was inadvertently created in the 1990's when two murine leukemia virus (MLV) genomes (pre-XMRV1 and pre-XMRV-2) recombined during passaging of the CWR22 tumor in mice. The conclusion that XMRV originated from mice and not the patient was based partly on the failure to detect XMRV in early CWR22 xenografts. While that deduction is certainly justified, we examined the possibility that a closely related virus could have been present in primary tumor tissue. Here we report that we have located the original prostate tumor tissue excised from patient CWR22 and have assayed the corresponding DNA by PCR and the tissue sections by fluorescence *in situ* hybridization for the presence of XMRV or a similar virus. The primary tumor tissues lacked mouse DNA as determined by PCR for intracisternal A type particle DNA, thus avoiding one of the limitations of studying xenografts. We show that neither XMRV nor a closely related virus was present in primary prostate tissue of patient CWR22. Our findings confirm and reinforce the conclusion that XMRV is a recombinant laboratory-generated mouse virus that is highly adapted for human prostate cancer cells.

## Introduction

The xenotropic murine leukemia virus-related virus (XMRV) is a gammaretrovirus discovered during studies of prostate cancer patients with a subtle genetic deficiency in the gene for the antiviral protein RNase L [1]. While several subsequent studies provided additional evidence for either XMRV or a closely related virus in prostate cancer patients [2]–[6], more studies either failed to detect any evidence of XMRV in prostate cancer patients or evidence was obtained but was limited to a small number of human samples [7]–[16]. Some of the positive findings in prostate cancer including, but not limited to, integration site mapping and detection of PCR products were later found to be the result of laboratory contamination [17], [18]. Possible sources of contamination include mouse DNA harboring MLV proviruses, XMRV plasmid or PCR products, and XMRV itself from infected cell lines. For instance, mouse DNA is sometimes present at trace amounts in some Taq polymerases, PCR master mix preparations and DNA extraction kits leading to false positives in PCR assays [19]–[22]. False negatives can also be produced in PCR assays further confounding detection of XMRV or related viruses [23]. A single study also showed the presence of XMRV in chronic fatigue syndrome patients [24], but recently those results have been largely attributed to laboratory contamination [25]–[27] and the original report was retracte [28]. Based on a recent large-scale study of blood donors in the US, it is unlikely that XMRV *per se* has entered this human population to any significant extent (0% prevalence; 95% confidence interval 0%–0.017%) [29]. Nevertheless, some of the positive findings involving non-PCR based methods, such as serology, immunohistochemistry and fluorescence *in situ* hybridization (FISH), that have seemingly detected XMRV or similar viruses in human samples have yet to be fully explaine [1], [3], [24]. Such evidence leaves open the possibility that either mouse DNA or an XMRV-like virus is present in at least some humans.

Against this backdrop, the origin of XMRV was recently elucidated by studying the human prostate cancer cell line, 22Rv1, and its xenograft precursors grown in nude mic [30]. The 22Rv1 cells are infected with, and produce high titers of, an XMRV that is nearly identical in sequence to XMRV strain VP62 from prostate cancer studie [1], [30], [31]. The origin of 22Rv1 cells can be traced back to a human prostate carcinoma (Gleason grade of 9) that was excised in 1992 at Case Western Reserve Universit [32]. Subsequently, the tumor, dubbed CWR22, was serially transplanted in nude mice. In 1996, after four years of serial passage in nude mice, castration of the mice was performed leading to regression and relapse of the tumo [33]. The resultant androgen-independent tumor, CWR22R, was then serially transplanted in mice until 1999 when it was used to establish the 22Rv1 cell lin [34]. High levels of XMRV are present in the 22Rv1 cell lines and in late passages of the CWR22 tumor, but not in early xenograft [30], [35]. Remarkably, the host mice contain two proviruses, pre-XMRV1 and pre-XMRV2, with long stretches (>3.2 kb) that are 99.9% identical to XMR [30]. It was hypothesized that recombination between the two proviruses led to XMRV and that XMRV was absent from the original tumor. However, the original tumor samples were not evaluated in those reports while xenografts inevitably contain low levels of mouse cells. The presence of endogenous mouse proviruses in the DNA of such contaminating mouse cells limits the choice of probes and PCR primers that could be used to uniquely identify XMRV-like elements in those samples. Here we describe the analysis of paraffin-embedded prostate blocks from patient CWR22 and show that neither XMRV nor closely related viruses are present in the primary tumor.

## Materials and Methods

### Processing of Prostate Tissue Blocks

Processing of formalin-fixed and paraffin-embedded (FFPE) blocks was performed in the Genomic Medicine Institute and Department of Laboratory Medicine, Cleveland Clinic. Five prostate tissue paraffin blocks from patient CWR22 (labeled A, B, C, E, and K) were sectioned at a width of 5 µ on a microtome that had been used exclusively for human samples. Sections were either collected in tubes for DNA extraction or placed on microscope slides for FISH analysis. Sections were stored in a 4°C refrigerator in the Genomic Medicine Institute biorepository (Cleveland Clinic). DNA extraction was performed in the same laboratory in which neither XMRV nor XMRV plasmid was ever used. The tissue collected originally from which the CWR22 transplant was produced was discarded tissue and no patient consent was required.

### Extraction of DNA from prostate sections

DNA extraction was performed by the following method (provided by Dr. Charis Eng, Genomic Medicine Institute, Cleveland Clinic: http://www.lerner.ccf.org/gmi/gmb/methods.php). Deparaffinization was done by adding 1 ml xylene to 18 sections (5 µ width each), shaking gently for 10 min, centrifugation for 10 min at 16,000 g at room temperature and discarding the supernatants. This step was repeated twice. The extraction was then performed with 1 ml each of 100% ethanol (2 times), 80% ethanol (2 times) and 50% ethanol (2 times), each time centrifuging for 10 min at 16,000 g at room temperature and discarding the supernatants. To the pellet 1 ml of nuclease-free water (USB/Affymetrix) was added and incubated at 4°C overnight. The pellet was collected after centrifugation for 10 min at 16,000 g at room temperature after discarding the supernatant. Nucleic Acid Lysis buffer, 700 µl, (10 mM Tris Base, 400 mM NaCl, 2 mM Na_2_EDTA and 0.7% SDS), was added to the pellet. Proteinase K, 50 µl (30 mg/ml) (Invitrogen) was added and digestion was performed at 65°C for 24 hrs. An additional 50 µl of proteinase K solution was added, incubated overnight at 65°C, 250 µl of 6 M NaCl was added, mixed thoroughly, and left at room temperature for 10 min. Samples were centrifuged for 10 min at 16,000 g at room temperature to pellet the DNA and supernatants were gently discarded. Pellets were washed with 70% ethanol and air dried on the bench top for a few min. Each pellet was resuspended in 40 µl TE (10 mM Tris-HCl pH 8.0; 1 mM EDTA) (USB/Affymetrix) and stored at 4°C.

### Single nucleotide polymorphism (SNP) genotyping (Roswell Park Cancer Institute)

SNP genotyping was performed using the MassARRAY Compact system (Sequenom, Inc., San Diego, CA) on a panel of 30 custom SNP assays designed using RealSNP and MassARRAY Assay Designer (Sequenom). Briefly, the protocol involves PCR amplification of 10 ng DNA using SNP specific primers, followed by a base extension reaction using the iPLEX Gold chemistry (Sequenom). The final base extension products were treated and spotted on a 384-pad SpectroCHIP (Sequenom) using a ChipSpotter LT nanodispenser (Samsung). A MassARRAY Analyzer Compact MALDI-TOF MS (Sequenom) was used for data acquisition from the SpectroCHIP. The resultant genotypes were called using MassARRAY Typer Analyzer v4.0 (Sequenom).

### Quantitative PCR (qPCR) for XMRV (Cleveland Clinic)

DNA samples were diluted to 100 ng/µl in TE buffer and 2 µl (200 ng) aliquots were used in duplicate for the qPCR assays (except for sample K, 17 ng of DNA was used due to a lesser amount of available DNA). Fast Mastermix (Applied Biosystems) was used for the qPCR assays using a Step One Plus Real time PCR machine following the manufacturer's instructions (Applied Biosystems). PCR conditions for using PCR Fast Mastermix were: 95°C for 20 sec for initial denaturation followed by 95°C for 1 sec, 60°C for 20 sec (data collection step), repeated for 50 times. The oligonucleotide probes contained 6-carboxyfluorescein (FAM) linked to the 5′ end and Nonfluorescent Quencher-Minor Grove Binder (NFQ-MBG) linked to the 3′ end (Applied Biosystems].

*env* gene:

6124F: 5′-GGCCGAGAGAGGGCTACT-3′

6159R: 5′-FAM-CACATCCCCATTTGCC-NFQ-MGB-3′

6197R: 5′-TGATGATGATGGCTTCCAGTATGC-3′

*gag* gene:

625F: 5′-GTAACTACCCCTCTGAGTCTAACCT-3′

668F: 5′-FAM-TCCAGCGCATTGCATC-NFQ-MGB-3′

708R: 5′-CTTCTTGACATCCACAGACTGGTT-3′

*pol* gene:

4843F: 5′-CGGGACAGAACTATCCAGTATGTGA-3′

4873F: 5′-FAM-ACCTGCACCGCCTGTG- NFQ-MGB-3′

4912R: 5′-TGGCTTTGCTGGCATTTACTTG -3′

As an internal control, we measured levels of the RNase P gene (a single-copy gene) encoding the RNA moiety for the RNase P enzyme. VIC-labeled control RNase P primer-probe combination from Applied Biosystems was used. A known copy number of the full length XMRV VP62 genome in plasmid pcDNA 3.1 [36] was used as a positive control to test each primer-probe combination.

### Intracisternal A-particles (IAP) qPCR assays (Cleveland Clinic)

QPCR for mouse IAP DNA was performed with the following oligonucleotide primers/probe:

IAP-1414F: 5′-TGGCGAAAGTCAGCGTACTG-3′

IAP-1435F: 5′-FAM-TCAACCTCCCGGCAGT-NFQ-MGB-3′

IAP-1472R: 5′-CATAGGGCGGACCTTGAAAC-3′

As a positive control for IAP, mouse tail DNA was extracted using Qiagen DNA extraction kit, its concentration measured by absorbance and serially diluted in TE buffer to generate the standard curve. PCR conditions were the same as those used to detect XMRV sequences.

### Real-time RT-PCR testing for XMRV (Abbott Molecular)

Single-round real-time reverse transcriptase polymerase chain reaction (RT-PCR) prototype assays were run on the *m*2000*rt* ^TM^ (Abbott Molecular Inc., Des Plaines, IL) instrument. An average of 500 ng of DNA from prostate cancer patient CWR22 (blocks A, B, C, E and K) was amplified with two primer sets designed to individually target the polymerase (*pol*) or envelope (*env*) regions of the XMRV genome. Each DNA sample diluted in water to achieve a 25 µl volume was combined with 25 µl of master mix that contained 10x EZ buffer, rTth enzyme, dNTPs, Rox reference dye, MnCl_2_, primers, and probes, to obtain a final PCR reaction volume of 50 µl. Primer/probe sequences, cycling conditions and the sensitivity/specificity estimation of *pol* and *env* RT-PCR assays have been described in detail previousl [37]. A primer/probe set for detecting the 136 bases of human *β*-globin gene was used to control for specimen adequacy and was amplified and detected simultaneously with XMRV (Fam signal) in the same reaction with a different fluorescence dye (Cy5 signal). TE buffer containing 1.5 μg/mL of poly dA:dT was used as assay negative control (NC). XMRV VP62 DNA plasmid diluted in the NC was used as assay positive control (PC).

### Fluorescence *in situ* hybridization (FISH) (Abbott Diagnostics)

The XMRV-SO FISH probe was prepared by directly labeling the entire plasmid DNA (∼13.6 kb) of clone VP62/pcDNA3.1 carrying a full-length genome (∼8.2 kb) of XMRV VP62 (36) with SpectrumOrange fluorophore through chemical reactions as described previousl [38], [39]. The percent incorporation of SpectrumOrange in the XMRV-SO probe was ∼8%. CEP8-SA probe derived from the centromeric sequence of human chromosome 8 and directly labeled with SpectrumAqua fluorophore was obtained from Abbott Molecular, Inc.

For evaluation of XMRV FISH probe performance, XMRV uninfected DU145 prostate cancer cell [36] were used as a negative control, while 22Rv1 prostate cancer cells harbouring ∼10 integrated copies of XMRV per cell and generating high–titer XMRV virus [31] were used as a positive control. Both cell lines were grown in DMEM-F12 complete medium (Invitrogen, Carlsbad, CA) at 37°C in the presence of 5% CO_2_. After reaching 60%–70% confluence, 1 ml of colcemid solution (10 µg/ml; Invitrogen) was added per 50 ml culture medium and cells were cultured at 37°C for 2 hr. Cells were harvested after trypsinization, washed once with 40 ml of 1x DPBS (Invitrogen), resuspended in 40 ml of 0.075 M potassium chloride solution (Invitrogen), and incubated at 37°C for 30 min. Cells were subsequently washed with 40 ml of Carnoy's fixative (3∶1 v/v methanol:glacial acetic acid; Fisher, Pittsburgh, PA) four times, resuspended in 5 ml of Carnoy's fixative and stored at −20°C. Slides with a mixture of DU145 and 22Rv1 were prepared by depositing 10 µl of each cell suspension on a SuperFrost Plus positively charged slide (ThermoShandon, Pittsburgh, PA). The slide was air-dried overnight prior to FISH pretreatment and hybridization.

Cell specimen slides were pretreated in 2x SSC (0.3 M NaCl, 0.03 M sodium citrate, pH 7.0; Invitrogen) at 73°C for 2 min then incubated in 0.5 mg/ml pepsin in 10 mM HCl (USB, Cleveland, OH) at 37°C for 10 min. Slides were rinsed in 1x DPBS (Invitrogen) for 5 min at room temperature, fixed in 1% neutral-buffered formalin solution (Fisher) for 5 min, then immersed in 1x DPBS for 5 min. Slides were dehydrated in an ethanol series of 70%, 85%, and 100% for 1 min each, and then air-dried. Ten µl of hybridization solution was prepared by mixing 100 ng XMRV-SO, 100 ng CEP8-SA, 1000 ng sonicated human placental DNA, 250 ng human Cot-1 DNA, and 7 µl LSI/WCP hybridization buffer (Abbott Molecular, Inc.), and was applied to each slide. A coverslip (22×22 mm; VWR, Radnor, PA) was placed over the probe solution, and sealed to the slide with rubber cement (Staples, Framingham, MA). Probes and cell nucleic acids on each slide were co-denatured at 73°C for 3 min and then hybridized at 37°C for 16–24 hrs on a hybridization platform (ThermoBrite; Abbott Molecular, Inc.). After hybridization, slides were washed in 0.4x SSC/0.3% NP-40 (Abbott Molecular, Inc.) for 2 min at 73°C and then in 2x SSC/0.1% NP-40 (Abbott Molecular, Inc.) for 1 min at room temperature. Ten µl of nuclear counterstain DAPI II (125 ng/ml; Abbott Molecular, Inc.) was applied to each specimen, and slides were evaluated under a fluorescence microscope. XMRV-SO probe was visualized with an orange filter set, CEP8-SA probe was visualized with an aqua filter set, and DAPI nuclear staining was visualized with a DAPI filter set.

Slides mounted with FFPE prostate cancer tissue sections were baked at 56°C for 4 hrs then stored at room temperature. In preparation for FISH hybridization, tissue specimen slides were deparaffinized three times in Hemo-De solvent (Scientific Safety Solvents, Keller, TX) for 5 min each at room temperature and rinsed in absolute ethanol twice for 1 min each. Slides were subsequently pretreated in a solution of 45% formic acid (Fisher)/0.3% hydrogen peroxide (Calbiochem, San Diego, CA) for 15 min at room temperature and rinsed in H_2_O for 3 min. Slides were then incubated in pretreatment solution (Abbott Molecular, Inc.) at 80°C for 35 min, washed in H_2_O at room temperature for 3 min, incubated in a pepsin solution (1.5 mg/ml in 0.1 N HCl) at 37°C for 22 min, rinsed in H_2_O at room temperature for 3 min. Slides were subsequently dehydrated in 70%, 85%, and 100% ethanol for 1 min each, and allowed to dry at room temperature. Ten µl of probe hybridization mix containing 100 ng XMRV-SO, 100 ng CEP8-SA, 1000 ng sonicated human placental DNA, 250 ng human Cot-1 DNA, and 7 µl LSI/WCP hybridization buffer was placed over each tissue section. A coverslip was applied and edges were sealed to the slide with rubber cement. Probes and tissue specimen nucleic acids on each slide were co-denatured for 5 min at 73°C and hybridized for 16–24 hr at 37°C on a ThermoBrite. After hybridization, slides were placed in 2x SSC/0.1% NP-40 at room temperature for 5–10 min, washed in 0.4 x SSC/0.3% NP-40 at 73°C for 2 min and in 2 x SSC/0.1% NP-40 at room temperature for 1 min. Ten µl of nuclear counterstain DAPI I (1,000 ng/ml; Abbott Molecular, Inc.) was applied to each tissue section, and slides were evaluated under a fluorescence microscope.

### Ethical Statement

These studies were approved by the Cleveland Clinic Foundation Institutional Review Board #1.

## Results

### Identification and verification of CWR22 prostate tissues

In 1992, prostate cancer patient CWR22 underwent transurethral resection of the prostate at Case Western Reserve Universit [32]. Following surgery, chips of prostate tissue were fixed in 10% neutral buffered formalin and embedded in paraffin blocks. Following diagnostic studies and issuance of a standard pathology report, the blocks were kept in storage by the Department of Pathology (Case Western Reserve University) at constant room temperature, mainly in unlighted rooms. In mid-2011, the tissue blocks were identified though archived hardcopy records as having originated from patient CWR22 and were then retrieved from storage. The University Hospitals (Cleveland) Institutional Review Board allows for the maintenance of patient and sample records for future studies; with an ability for re-linkage while maintaining a firewall to prevent release of any public health information to investigators.

The prostate blocks were sectioned on a microtome used exclusively for human tissues in the Department of Laboratory Medicine (Cleveland Clinic). The DNA was extracted in the Genomic Medicine Institute (Cleveland Clinic) in a laboratory where neither XMRV nor XMRV nucleic acids were used. To confirm the common origin of the specimens, the DNA samples from five FFPE prostate blocks from patient CWR22 (labeled as A, B, C, E & K) were compared among themselves as well as to the previously described [35] samples from a CWR xenograft and 22Rv1 cell line by single nucleotide polymorphism (SNP) analysis at Roswell Park Cancer Institute (Buffalo). We relied on a method of detecting SNPs using the fully automated system from Sequenom, Inc. (San Diego, CA). The system is based on PCR-amplification of the region of interest, followed by primer extension through the polymorphic site in the presence of three deoxyribonucleotide triphosphates and one dideoxyribonucleotide triphosphate, and determination of the nucleotide composition of the short extension products using mass-spectrometry [40]. We observed that all the seven samples carried an identical pattern of SNPs in all thirty of the examined sites (Table 1), thus confirming that the prostate tissue blocks originated with the same patient as did the CWR xenograft and 22Rv1 cells.

**Table 1:** SNP Genotyping of CWR22 primary tumor, CWR22 xenograft, and 22Rv1 cells.

SNP ID	Detected allele
rs10083901	A
rs1080169	C
rs10853605	C
rs1397266	AG
rs1559806	CT
rs16952692	C
rs16952847	A
rs16953030	A
rs17736674	A
rs17743658	A
rs1789223	C
rs2027735	T
rs2282543	T
rs2298617	G
rs2442962	G
rs2584076	T
rs35952031	G
rs3764466	G
rs4128208	C
rs4390682	A
rs4456603	A
rs4711374	C
rs608986	G
rs61751988	G
rs620898	T
rs7229495	G
rs7235543	T
rs7238500	A
rs75667697	T
rs77386888	A

### Absence of XMRV DNA or that of a closely related virus in patient CWR22

To determine if nucleic acids from XMRV or a closely related virus was present in the prostate of patient CWR22, PCR was independently performed in the Department of Cancer Biology, Cleveland Clinic (Cleveland) and at Abbott Molecular, Inc. (Des Plaines).

#### qPCR at Cleveland Clinic

**Figure 1:**
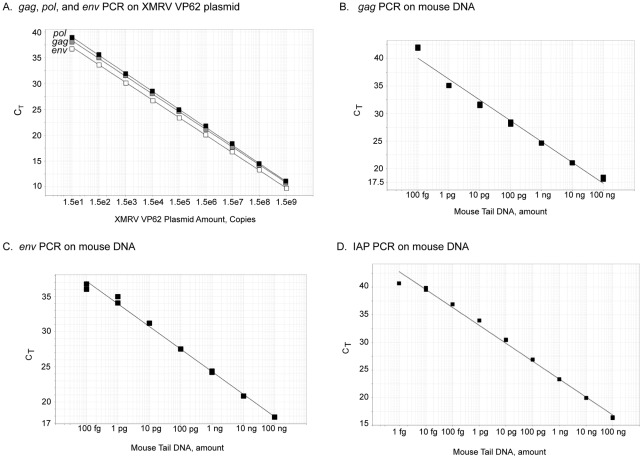
The sensitivity and specificity of qPCR assays were demonstrated with linear regression curves for XMRV VP62 plasmid and mouse tail DNA. (A) Nine different dilutions of XMRV VP62 plasmid (15 to 15×10^9^ copies each reaction in duplicate) were used to generate the standard curve using *gag*, *pol* and *env* primer probe combinations. (B–D) Serial dilution of mouse tail DNA (1 fg to 100 ng each reaction in duplicate) were used to generate the data for (B) *gag*, (C) *env* and (D) IAP. e, exponent (10 to the power of *n*).

To determine the sensitivities of qPCR for XMRV *gag*, *pol*, and *env*, assays were done with the full-length viral molecular clone, plasmid XMRV VP62 [36]. As few as 15 copies of XMRV plasmid were reproducibly detected with primers and probes for all three XMRV genes (*gag*, *pol*, and *env*)(Fig. 1A). Because the nucleotide sequence of XMRV is up to 95% identical with several MLV endogenous proviruse [1], we sought to determine if qPCR for XMRV *gag*, *pol*, and *env* would also amplify MLV sequences from mouse DNA. QPCR with XMRV *gag* and *env* primers did amplify products from as little as 100 fg of mouse tail DNA, whereas the XMRV *pol* primers did not produce PCR products from mouse DNA (Fig. 1B&C and data not shown). These results suggest that MLV endogenous proviruses can be detected by qPCR with either the XMRV *gag* or *env* primers, but not with the *pol* primers. To monitor for mouse DNA contamination, qPCR was performed for mouse IAPs (endogenous retrovirus-like mobile elements [41] that are readily detectable by PC [18]). Remarkably, as little as 1 fg of mouse tail DNA was detected by qPCR for IAP DNA (Fig. 1D). These results are consistent with the presence of about 50 MLV proviruses [42] and 1000 copies of IAP DNA per mouse haploid genom [41].

**Figure 2:**
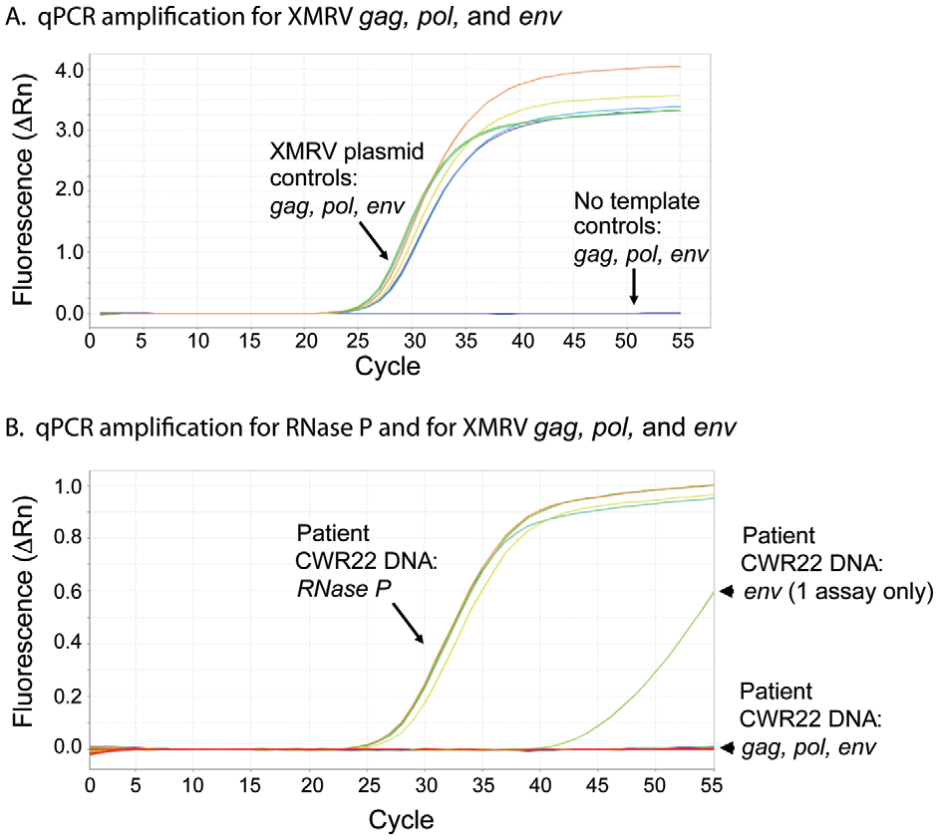
An absence of detectable levels of XMRV DNA or closely related DNA in CWR22 primary prostate tissues was determined by qPCR analysis. Amplification plot of real-time qPCR analysis for the (A) detection of XMRV specific regions (*gag*, *pol* and *env*) using XMRV VP62 plasmid DNA (3,750 copies) and (B) in DNA extracted from different sections of CWR22 prostate tissues (tissue blocks A, B, C & E, each assayed in duplicate). For block C only, 1 of 2 assay for *env* was weakly positive, all other assays for *gag*, *pol* and *env* were negative. *RNase P* probes were used to detect the presence of genomic DNA in tumor tissues.

To demonstrate representative fluorescence units as a function of cycle number, XMRV VP62 plasmid was subjected to qPCR for XMRV *gag*, *pol*, and *env* in comparison to control reactions lacking added DNA (Fig. 2A). QPCR assay were performed using DNA from the different prostate tissue blocks from patient CWR22. However, no XMRV DNA was detected in duplicate assays for all three XMRV genes with CWR22 prostate DNA from blocks A, B, C, E and K (Fig. 2B and Table 2). One of 2 assays for XMRV *env* in block C only produced a weak response at >40 cycles, which is below the reliable limit of detection and likely represents an artifact. No mouse IAP DNA was detected by PCR of the DNA extracted from the CWR22 prostate tissues indicating an absence of contaminating mouse DNA in these samples (Table 2).

**Table 2:** Summary of XMRV and Control Assays in CWR22 Prostate Samples.

Prostate Block of Patient CWR22	A	B	C	E	K
PCR-Cleveland Clinic (XMRV *gag*)	−, −	−, −	−, −	−, −	−, −*
PCR-Cleveland Clinic (XMRV *pol*)	−, −	−, −	−, −	−, −	−, −*
PCR-Cleveland Clinic (XMRV *env*)	−, −	−, −	−, +/−**	−, −	−, −*
PCR-Cleveland Clinic (RNase P)	+	+	+	+	+
PCR-Cleveland Clinic (IAP)	−	+/−***	−	−	−
PCR-Abbott (XMRV *pol*)	−	−	−	−	−
PCR-Abbott (XMRV *env*)	−	−	−	−	−
PCR-Abbott (β-globin)	+	+	+	+	+
FISH-Abbott (XMRV)	−	−	−	−	−
FISH-Abbott (CEP8)	+	+	+	+	+

* Reduced input DNA amount to 17 ng, **Ct value of 45, *** Ct value of 41.

#### Real-time RT-PCR at Abbott Molecular

**Figure 3:**
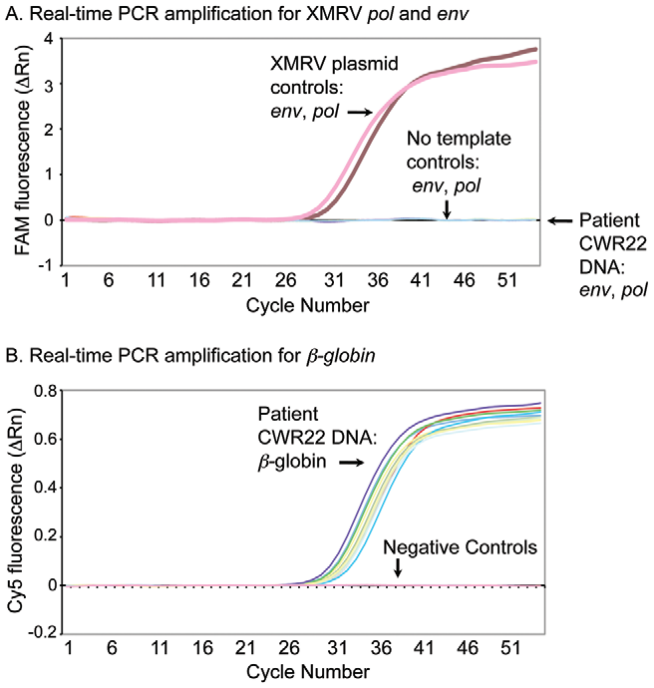
An absence of detectable levels of XMRV DNA or closely related DNA in CWR22 primary prostate tissues was determined by real-time PCR analysis. Amplification plots from the real-time PCR analysis of (A) XMRV (FAM) signal in DNA extracted from different sections of CWR22 prostate tissues and run controls with the *pol* and *env* primer/probe sets; (B) *β-*globin (Cy5) signal during the same run.

To further interrogate the prostate tissue specimens for evidence of XMRV infection, two additional single-round real-time RT-PCR assays targeting XMRV *pol* and *env* were utilized. Sensitivity and specificity of the two assays for detection of XMRV have previously been demonstrated; these were based on comparison to multiple assays with coded control panels created by the Blood XMRV Scientific Research Working Group (BSRWG) [37], [43]. Using whole blood and plasma panels prepared by the BSRWG, these assays were equal to the most sensitive assays teste [43]. Using serial dilutions of the XMRV VP62 plasmid controls, both assays could reliably detect 5 copies of DNA per reaction. Based on this sensitivity of the assay, we estimate a lower limit of detection of about 1 proviral genome per 17,000 cells. Positive control reactions were positive and negative controls were negative (Fig. 3A). No XMRV was detected by either the *pol* or *env* assays in DNA extracted from CWR22 prostate blocks A, B, C, E and K (Fig. 3A; Table 2). Signal amplification plots of *β-*globin (Cy5) amplified during the same run (Fig. 1B, Table 2) revealed that all patient samples were positive for *β*-globin DNA, indicating there was sufficient DNA present in the samples for amplification.

### XMRV FISH analysis of CWR22 tissue sections

**Figure 4:**
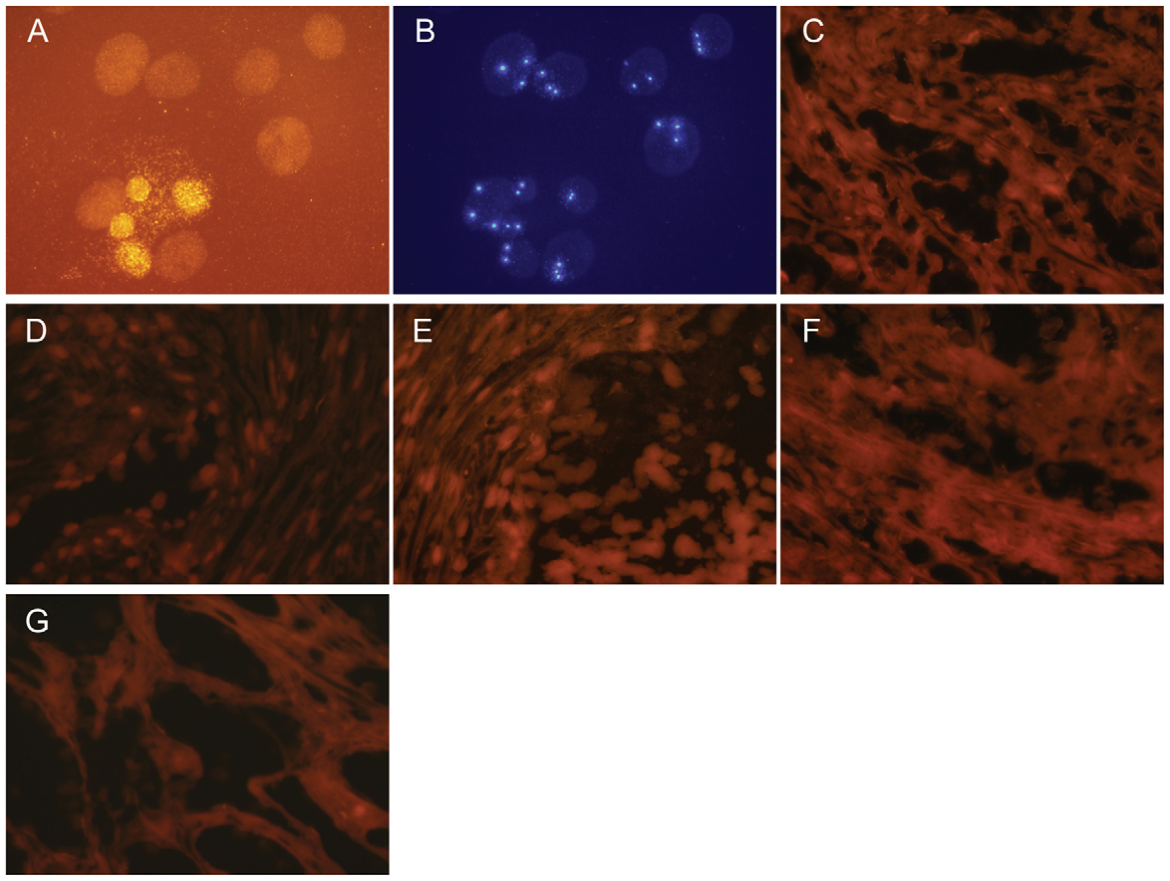
FISH analysis did not detect XMRV nucleic acid or closely related sequences in CWR22 primary prostate tissues. Each slide was hybridized with a probe mix consisting of XMRV-SO viral probe derived from a full-length XMRV VP62 and CEP8-SA internal control probe from the centromeric sequence of human chromosome 8. (A) representative image showing XMRV-SO orange staining in a mixture of uninfected DU145 prostate cancer cells and XMRV-infected 22Rv1; (B) the same image showing CEP8-SA aqua staining in DU145 (three copies/cell) and 22Rv1 (two copies/cell); (C – G) representative images showing XMRV-SO FISH results on tissue sections from blocks A, B, C, E, and K, respectively, from CWR22.

An alternative approach for molecular identification of viral infection is FISH. FFPE tissue sections from each of the CWR22 prostate blocks A, B, C, E and K were screened for evidence of XMRV infection using a directly-labeled probe (XMRV-SO). The probe mix also contained a second probe, CEP8-SA, which hybridizes to the centromeric region of human chromosome 8 that served as an internal control to monitor the integrity of the FISH hybridization step. Slides containing a mixture of uninfected DU145 prostate cancer cells and XMRV-infected 22Rv1 prostate cancer cells (≥10 integrated copies/cell) were used to establish the specificity and localization of FISH hybridization. Results of this analysis are shown (Fig. 4A & B). The CEP8-SA chromosomal marker readily distinguished the two cell lines as three copies were present in DU145 whereas 22Rv1 contained two copies. XMRV FISH hybridization was only observed for the 22Rv1 cells. XMRV-staining in 22Rv1 cells was primarily localized to the nucleus, while some staining was found in the cytoplasm. Pretreatment of the cells with RNase A to digest both cellular and viral RNA prior to hybridization of the XMRV-SO probe resulted in a punctuate pattern of staining, indicative of integrated XMRV proviral DNA, localized to the nucleus (data not shown). Representative images of the XMRV FISH analysis on CWR22 tissue sections from blocks A, B, C, E and K are shown (Fig. 4C–G, respectively). The tissue sections from blocks B, C and E were negative for staining with the XMRV-SO probe although they were positive for the internal control CEP8-SA probe (Fig. 4 and data not shown). Sections from blocks A and K were negative for XMRV staining with the exception of some cells along one edge of each slide. To examine specificity of this staining, a human papilloma virus probe type 16 probe labeled in the same manner as the XMRV probe was hybridized to sections from blocks A and K. Similar to what was observed with the XMRV-SO probe, the sections were negative with the exception of cells along the same edge of the slides (data not shown). Thus, the staining observed along the edge of these slides appears to be a non-specific artifact. Based on this analysis, we conclude that the sections from all of the CWR22 tissue blocks are negative for XMRV and related viruses.

## Discussion

A previous study proposed that XMRV was generated by recombination between two endogenous proviruses of mice, pre-XMRV1 and pre-XMRV2, during passage of the CWR22 tumor cells in nude mic [30]. While XMRV originated in mice, it is highly adapted for human prostate epithelial cells as a result of virus-host cell interactions in vivo. For instance, XMRV trafficked to prostatic epithelium within 6 or 7 days of experimental infection of rhesus macaque [44], although not in pigtailed macaques at 119 days post-infectio [45]. Initial infections in the CWR22 cell lineage that led to the 22Rv1 cell line were likely facilitated by innate immunity deficiencies. Interestingly, the 22Rv1 cells are homozygous for the same reduced activity variant of RNase L (R462Q) as some prostate cancer patients in the original XMRV stud [1], [31]. We confirmed that the primary prostate tissue from patient CWR22 is QQ for RNase L by genotyping analysis (data not shown). There is also a deficiency in the host restriction factor APOBEC3G in 22Rv1 cells and other prostate cancer cell line [46], [47]. In addition, androgen stimulates viral transcription and replication due to the presence of a glucocorticoid response element (GRE) in the U3 region of the XMRV LTR [48], [49]. Therefore, androgen may have stimulated XMRV infection of CWR22 cells during passage in male mice. Also, XMRV may have contributed to the growth of the CWR22 tumors in mic [50].

A limitation of using mouse xenografts to determine the origin of XMRV is the inevitable presence of low levels of mouse cells and DNA. We have avoided this complication by studying primary tissue from the patient as demonstrated by an absence of mouse IAP sequences. In the current study, five sensitive real-time PCR assays targeting XMRV *gag*, *pol* and *env* were utilized to screen for the presence of XMRV in CWR22 prostate cancer tissue. None of the assays detected XMRV in DNA extracted from five tissue blocks. Of note, the PCR primers/probe combinations for XMRV *gag* and *env* were capable of amplifying sequences from mouse DNA but failed to detect evidence of MLV infection in the prostate of patient CWR22 (with sensitivities as low as 1 viral genome per 17,000 cells). Similarly, FISH using XMRV DNA as probe failed to detect viral nucleic acid in the CWR22 tissue. Our findings conclusively show an absence of XMRV or related viruses in prostate of patient CWR22, thereby strongly supporting a mouse origin of XMRV. While XMRV was originally identified in a study of prostate cancer patients [1], the sequence of XMRV present in 22Rv1 cells [30] is virtually identical with XMRV cloned using human prostate samples, thus suggesting laboratory contamination with XMRV nucleic acid from 22Rv1 cells as the source. Further experiments designed to confirm or refute this hypothesis are currently underway.
